# Supplementary material for: HBV seroprevalence and liver fibrosis status among population born before national immunization in Southern Thailand: Findings from a health check-up program
Source: PLoS One. 2022 Jun 24;17(6):e0270458. doi: 10.1371/journal.pone.0270458 (PMC9231792; doi:10.1371/journal.pone.0270458)
Supplement: Attachment — Submitted filename: Response to reviewers.docx [file pone.0270458.s001.docx]

Journal Requirements:

When submitting your revision, we need you to address these additional requirements.

1. Please ensure that your manuscript meets PLOS ONE's style requirements, including those for file naming. The PLOS ONE style templates can be found at

https://journals.plos.org/plosone/s/file?id=wjVg/PLOSOne_formatting_sample_main_body.pdf and

https://journals.plos.org/plosone/s/file?id=ba62/PLOSOne_formatting_sample_title_authors_affiliations.pdf

*- Thank you, we have edited the format of the entire manuscript according to the PLOS ONE’s style.*

2. We note that Figure 2 in your submission contain [map/satellite] images which may be copyrighted. All PLOS content is published under the Creative Commons Attribution License (CC BY 4.0), which means that the manuscript, images, and Supporting Information files will be freely available online, and any third party is permitted to access, download, copy, distribute, and use these materials in any way, even commercially, with proper attribution. For these reasons, we cannot publish previously copyrighted maps or satellite images created using proprietary data, such as Google software (Google Maps, Street View, and Earth). For more information, see our copyright guidelines: http://journals.plos.org/plosone/s/licenses-and-copyright.

We require you to either (1) present written permission from the copyright holder to publish these figures specifically under the CC BY 4.0 license, or (2) remove the figures from your submission:

a. You may seek permission from the original copyright holder of Figure 2 to publish the content specifically under the CC BY 4.0 license.

We recommend that you contact the original copyright holder with the Content Permission Form (http://journals.plos.org/plosone/s/file?id=7c09/content-permission-form.pdf) and the following text:

“I request permission for the open-access journal PLOS ONE to publish XXX under the Creative Commons Attribution License (CCAL) CC BY 4.0 (http://creativecommons.org/licenses/by/4.0/). Please be aware that this license allows unrestricted use and distribution, even commercially, by third parties. Please reply and provide explicit written permission to publish XXX under a CC BY license and complete the attached form.”

Please upload the completed Content Permission Form or other proof of granted permissions as an "Other" file with your submission.

In the figure caption of the copyrighted figure, please include the following text: “Reprinted from [ref] under a CC BY license, with permission from [name of publisher], original copyright [original copyright year].”

b. If you are unable to obtain permission from the original copyright holder to publish these figures under the CC BY 4.0 license or if the copyright holder’s requirements are incompatible with the CC BY 4.0 license, please either i) remove the figure or ii) supply a replacement figure that complies with the CC BY 4.0 license. Please check copyright information on all replacement figures and update the figure caption with source information. If applicable, please specify in the figure caption text when a figure is similar but not identical to the original image and is therefore for illustrative purposes only.

The following resources for replacing copyrighted map figures may be helpful:

USGS National Map Viewer (public domain): http://viewer.nationalmap.gov/viewer/

The Gateway to Astronaut Photography of Earth (public domain): http://eol.jsc.nasa.gov/sseop/clickmap/

Maps at the CIA (public domain): https://www.cia.gov/library/publications/the-world-factbook/index.html and https://www.cia.gov/library/publications/cia-maps-publications/index.html

NASA Earth Observatory (public domain): http://earthobservatory.nasa.gov/

Landsat: http://landsat.visibleearth.nasa.gov/

USGS EROS (Earth Resources Observatory and Science (EROS) Center) (public domain): http://eros.usgs.gov/#

Natural Earth (public domain): http://www.naturalearthdata.com/

*- Thank you. We have removed figure 2 showing map figure, replaced it with simple figure instead.*

3. Please review your reference list to ensure that it is complete and correct. If you have cited papers that have been retracted, please include the rationale for doing so in the manuscript text, or remove these references and replace them with relevant current references. Any changes to the reference list should be mentioned in the rebuttal letter that accompanies your revised manuscript. If you need to cite a retracted article, indicate the article’s retracted status in the References list and also include a citation and full reference for the retraction notice.

*- We have reviewed the reference and change the reference style according to the journal’s style*

Additional Editor Comments:

This is a cross-sectional study of HBV and liver fibrosis in southern Thailand.

This is a well-written epidemiologic study. While the findings are not entirely unexpected, the methods are appropriate and the results informative. Additional clarifications would strengthen the manuscript further, including:

Was HCV or HIV serostatus considered?

Additional study limitations include the lack of HBV DNA quantification.

*- Thank you, editor, we have added the limitation statement regarding HCV, HIV, and HBV DNA tests in the discussion part.*

In Table 2, it would be helpful to stratify FIB-4 scores into low / high (>1.45 etc).

*-The proportions of each FIB-4 category had been added to table 2 already.*

Reviewers' comments:

5. Review Comments to the Author

**Reviewer #1:** The English language should be revised and improved throughout the manuscript.

*- We have carefully re-read the entire manuscript and made language editing and polishing thoroughly. Thank you for pointing out.*

Abstract (and Manuscript):

The Authors should explain how liver cirrhosis was defined by ultrasound, and which morphologic features were considered suggestive of liver cirrhosis.

*- The diagnosis of cirrhosis using ultrasound findings was explained in a further detail in material and methods part.*

The Authors state that HBsAg-antiHBc+ indicate past HBV infections. However, even though in most cases the HBsAg-anti-HBc+ pattern does indicate past, resolved infection, this pattern can also indicate false-positive anti-HBc, low level chronic HBV infection and even resolving acute infection. These possibilities should be mentioned for the 17.5% of patients in this group who did not have concomitant anti-HBs positivity.

*- Thank you, we have added the statement regarding this issue onto the results part as “All HBsAg+ patients (100%) were also positive for anti-HBc, indicating true HBV infection status. While 82.5% (95%CI: 78.8-85.7%) of patients with HBsAg-antiHBc+ also had anti-HBs positivity, reflecting that past HBV infection is the vast majority status of the patients with HBsAg-antiHBc+, whereas the remaining 17.5% (95%CI: 14.3-21.2%) who were negative for anti-HBs might be from either low level of anti-HBs, low HBsAg level, or false-positive anti-HBc.”*

Abstract:

Lines 40-41: ''One-fourth of past HBV patients had FIB-4>1.45 which correlates with significant fibrosis and increased HCC risk''.

FIB-4 < 1.45 can rule-out advanced fibrosis, but only at > 3.25 can rule-in advanced fibrosis; this should be clarified. In addition, only HBsAg carriers with FIB-4 > 2.2-2.4 have an increased HCC risk, and this also should be rectified.

*- Thank you, we made a better clarification regarding FIB-4 strata on the presence of fibrosis level as well as for HCC risk. The correction had been made to the material and methods part as “the cut-off values for determining significant fibrosis (F2 or higher) recommended by the World Health Organization (WHO) as follow: FIB-4 of <1.45 is a low threshold to exclude significant fibrosis, FIB-4 of 1.45-3.25 demonstrates indeterminate significant fibrosis status, and FIB-4 >3.25 is the high cut-off level to rule-in significant fibrosis.[11]”, and for HCC risk in the discussion part as “Furthermore, using the FIB-4 score as a noninvasive marker of liver fibrosis which has been validated in HBV patients[16], an increased risk of having significant liver fibrosis (F2 or higher) were observed not only in patients with current HBV infection (20.41%), but also in patients with past HBV infection (24.15%) when compared with patients without HBV infection (12.9%). Not only being a determinant of liver fibrosis status, FIB-4 had also been reported to be an independent predictor of HCC occurrence in patients with HBV. The FIB-4 cut-offs for an increased risk of HCC development varied among studies, from >1.29[17], >2.4[18], to >3.25[19]”.*

Discussion:

Lines 188-190: ''And eighty-two percent of HBsAg-antiHBc+ patients were also positive for anti-HBs, most of them had had a past HBV infection, thus increasing the risk of cirrhosis and the development of HCC''. The Authors should clarify on the basis of which evidence past HBV infection increases the risk of cirrhosis and the development of HCC.

Lines 213-214: ''and HCC surveillance is still recommended even in patients with HBV who turned to HBsAg seroconversion (antiHBc+, antiHBs+)''. The Authors refer to a 2011 article for this statement. They should provide much more recent evidence that surveillance is recommended nowadays.

*- For the discussion part, we have made a clarification on the role of past HBV infection on HCC development and added more recent references as “ Patients with past HBV infection, on the other hand, were thought to be at a low risk of long-term liver-related complications. Nonetheless, they were not as safe as patients without HBV infection, as mentioned earlier, 1.07% of them already had cirrhosis. And those with a high FIB-4 score might be at a higher risk of developing HCC in the future, even though the FIB-4 cutoff for determining HCC risk in patients with past HBV infection is yet to be determined. Patients with past HBV infection (HBsAg-antiHBc+) were also recently demonstrated to be associated with a higher risk of HCC in patients with non-alcoholic fatty liver disease[23], and other chronic liver diseases.[24] Moreover, there was a study reported that positive anti-HBc was observed in >75% of non-HBV, non-HCV HCC new cases in an HBV-endemic area,[25] suggesting that past HBV infection potentially linked to HCC development and those with past HBV infection might benefit from HCC surveillance. [26, 27]”*

Table 1: Marital status, creatinine and ALP should be removed, as irrelevant.

*- We have removed those parameters as suggested*

The data on gallstones are not relevant and should be removed.

The Authors should explain what ‘’nodules’’ mean. Does it mean surface nodularity? If so, why is there a discrepancy with liver cirrhosis? And why 61 cases in 49 patients are indicated (this is clearly wrong, and the 3.6 percentage is also wrong).

*- The hepatic nodule(s) means focal liver lesions not nodularity of the liver, we have clarified this on the result part. And removal of gallstones findings from the table has been done.*

**Reviewer #2:** This well written article summarizes a cross-sectional analysis of electronic medical records to understand the frequency of hepatitis B virus (HBV), current and past infections, and HBV’s association with advanced liver diagnostic tests usage and their findings. A total of 1690 Thai citizens living in the south, who had a qualifying health examination at one medical center, were included. The findings are consistent with prior studies in Thailand and regionally, both on HBsAg+ prevalence and the lack of awareness of the infection. The authors argue that providing more information on those with HBsAg- but anti-HBc+ is valuable as this group could have their HBV infection reactivated by some therapies. While the study’s findings are not particularly novel, they have value in providing a current understanding of HBV status as elimination efforts continue.

Major comment

The key issue that needs to be addressed is who was eligible for the study. Specifically, who selected packages E and F for the health assessment? What proportion of all health assessments at the medical center are E or F; what proportion of the population have health assessments elsewhere, etc. Could the estimates be biased away from the null (i.e., slight overestimates)? If so, then several edits would be in order: take the word “population” out of the title, mention this eligibility in the abstract, qualify the key finding and add information to the limitations.

*- Thank you for the valuable comment, ones who chose package E and F were accounted for one-third of all health check-up individuals. We have added the statement regarding this issue in the limitation part as “In addition, as the different check-up packages comes with different costs, those who chose to get packages which included HBV serology and ultrasonography may have a higher socioeconomic status than those who chose health check-up packages at a lower cost (the patients included in this study were accounted for one-third of all patients sought for health check-up at our center during the study period). Although a recent meta-analysis demonstrated that the low-income status may increase risk of HBV seroprevalence, the sensitivity analysis of patients residing in Asia in that meta-analysis showed no significant association between the low-income status and HBV seroprevalence.[32] A prior study in Thailand also showed that the HBsAg seroprevalence was not statistically significant higher in individuals who had income <10,000 baht/month (approximately 300 USD) compared to those who had higher monthly income.[33] Therefore, the prevalence of current HBV infection in our cohort might be underestimated as only those who voluntarily selected the higher cost packages were included, but we think it should not be considerably statistically significant.”*

Details

Abbreviations: it would be helpful to define the terms used in the abstract and the first usage in the manuscript.

Confidence intervals for prevalence estimates would be helpful to readers.

Consider using the term “proportion” in place of “rate.”

170 “health check-up in Thailand” Add ‘southern’ Thailand

231 “However, we included all patients from all 14 provinces in 232 Southern Thailand who sought health check-ups at our center during the study period,” Actually, only those asking for specific packages.

*- Thank you. We have made corrections throughout the manuscript as per the reviewer’s suggestion.*

Is there any history of alcohol documented in the medical records? It seems that this may be a limitation given alcohol’s association with liver disease.

*- Alcohol intake was not universally recorded; we have added this a one of the limitations of our study in the discussion part. Thank you.*
