# Supplementary material for: HBV seroprevalence and liver fibrosis status among population born before national immunization in Southern Thailand: Findings from a health check-up program
Source: PLoS One. 2022 Jun 24;17(6):e0270458. doi: 10.1371/journal.pone.0270458 (PMC9231792; doi:10.1371/journal.pone.0270458)
Supplement: Attachment — Submitted filename: Response to reviewers_R2.docx [file pone.0270458.s002.docx]

Responses to reviewers:

Additional Editor Comments:
Please address the minor revisions raised by the reviewer and have your manuscript reviewed carefully by a native English speaker and/or a professional editing service prior to resubmission.

- Thank you for your comments, we have revised according to the reviewer’s comments. And we also have a proofread by a professional editing service, the certificate of English editing is also submitted in the platform.

Reviewers' comments:

Reviewer's Responses to Questions

**Comments to the Author**

5. Is the manuscript presented in an intelligible fashion and written in standard English?

PLOS ONE does not copyedit accepted manuscripts, so the language in submitted articles must be clear, correct, and unambiguous. Any typographical or grammatical errors should be corrected at revision, so please note any specific errors here.

Reviewer #1: No

- Thank you, in this revised manuscript, we have a proofread by a professional editing service, the certificate of English editing is also submitted in the platform.

6. Review Comments to the Author

Please use the space provided to explain your answers to the questions above. You may also include additional comments for the author, including concerns about dual publication, research ethics, or publication ethics. (Please upload your review as an attachment if it exceeds 20,000 characters)

Reviewer #1: The manuscript has been considerably improved. However, the English is not entirely up to standard yet.
In addition, the statement "....whereas the remaining 17.5% (95%CI: 14.3-21.2%) who were negative for anti-HBs might be from either low level of anti-HBs, low HBsAg level, or false-positive anti-HBc" is not ideal. It should read : ""....whereas the remaining 17.5% (95%CI: 14.3-21.2%) who were negative for anti-HBs could have past, resolved infection but could also have falsely positive anti-HBc results or low level chronic HBV infection or resolving acute infection".

- Thank you, the correction has been made accordingly as follows: “whereas the remaining 17.5% (95%CI: 14.3-21.2%) who were negative for anti-HBs could have past, resolved infection but could also have falsely positive anti-HBc results, or low- level chronic HBV infection, or resolution of acute infection” (Results part: line 202-205).
